# Textile Based Electrochromic Cells Prepared with PEDOT: PSS and Gelled Electrolyte

**DOI:** 10.3390/s20195691

**Published:** 2020-10-06

**Authors:** Carsten Graßmann, Maureen Mann, Lieva Van Langenhove, Anne Schwarz-Pfeiffer

**Affiliations:** 1 Research Institute for Textile and Clothing, Niederrhein University of Applied Sciences, 41065 Mönchengladbach, Germany; frau.mann88@gmail.com (M.M.); anne.schwarz-pfeiffer@hs-niederrhein.de (A.S.-P.); 2 Center for Textile Science and Engineering, Ghent University, 9052 Ghent, Belgium ; Lieva.VanLangenhove@UGent.be

**Keywords:** smart textile, flexible device, passive display, multilayer matrix structure, gelatin electrolyte, alginate

## Abstract

Electrochromic devices can act as passive displays. They change their color when a low voltage is applied. Flexible and bendable hybrid textile-film electrochromic devices with poly-3,4-ethylenedioxythiophene polystyrene sulfonate (PEDOT:PSS) were prepared on polyethylene polyethylene terephthalate (PEPES) membranes using a spray coating technique. The electrolyte consisted of a gelatin glycerol mixture as host matrix and calcium chloride. Titanium dioxide was used as an ion storage layer and a carbon containing dispersion was used for the counter electrode on a polyester rip-stop fabric. The sheet resistance of PEDOT:PSS on PEPES was 500 Ohm/sq. A 5 × 5 electrochromic matrix with individually addressable pixels was successfully designed and assembled. The switching time of the pixels was 2 s at a voltage of 2.0 V directly after assembling. The use of titanium dioxide as ion storage also increased the contrast of the dark-blue reduced electrochromic layer. Coloration was not self-sustaining. The PEDOT:PSS layer needed a constant low voltage of at least 0.5 V to sustain in the dark-blue reduced state. The switching time increased with time. After 12 months the switching time was ~4 s at a voltage of 2.8 V. The addition of glycerol into the electrolyte extended the lifetime of a non-encapsulated textile electrochromic cell, because moisture is retained in the electrolyte. Charge carriers can be transported into and out of the electrochromic layer.

## 1. Introduction

Reversible color changing materials can be grouped according to the external stimulus, which induces the color change. For instance, hydrochromic materials react to the presence of water or water vapor, thermochromic materials react to the change of temperature, mechanochromic materials to mechanical stress, photochromic materials to illumination, and electrochromic materials to a voltage [1,2,3,4]. Electrochromic materials have been considered for decades. Color changing materials can not only be used as decorative elements, but they can also act as actuators. Electrochromic materials change their color reversibly depending on their current redox state. Prominent examples are electrochromic windows for buildings and rear-view mirrors in automotive applications [5]. 

Generally, the following performance parameters for electrochromic materials are of interest for potential applications: electrochromic contrast, coloration efficiency, write-erase efficiency, switching speed, stability, cycle life, and optical memory [6]. The electrochromic contrast or contrast ratio characterizes the transmittance of the electrochromic material in its bleached (colorless) state to the transmittance in the colored state. The ratio of the absorbance change to the charge carriers injected per electrode area is described by the coloration efficiency. The percentage of the originally formed colored state that subsequently can be electrochemically bleached is defined as the write–erase efficiency. The switching speed, also referred to as response time in some instances, is generally described as the time an electrochromic material requires for some fraction of the color to form or become bleached. The electrochromic stability is generally associated with the electrochemical stability. The degradation of the active redox couple leads to the loss of electrochromic contrast, and therefore the performance of the electrochromic material. The cycle life describes the number of write–erase cycles that can be performed whilst the material remains stable and without a noticeable decrease in performance. Optical memory means the materials can retain the new electrochromic state, when the redox state has been switched, meaning no further charge injection needs to be provided.

In electrochromic devices, a persistent but reversible color change is induced by an electrochemical oxidation or reduction process [7,8]. Materials which undergo an electrochromic color change can be inorganic or organic compounds. A widely studied and probably the most prominent material is tungsten(VI) oxide [9]. Another inorganic electrochromic material is iron(III) hexacyanoferrate(II), more commonly known as Prussian Blue [10]. The color change in electrochromic cells is possible at low voltages between 1 and 5 V [11,12,13]. The exact value depends on the redox potential of the electrolyte and the electrochromic material, which are used. The color change sustains in most cases only when the device is supplied with a low voltage. However materials with a memory-effect exist. These materials do not need a constant power supply to sustain the oxidized or reduced state [14]. Compared to light emitting devices like electroluminescent and (organic) light emitting devices, which must be powered continuously, electrochromic devices consume less power and are economically favored. The color impression and the contrast are independent of the observation angle and surrounding brightness.

**Figure 1:**
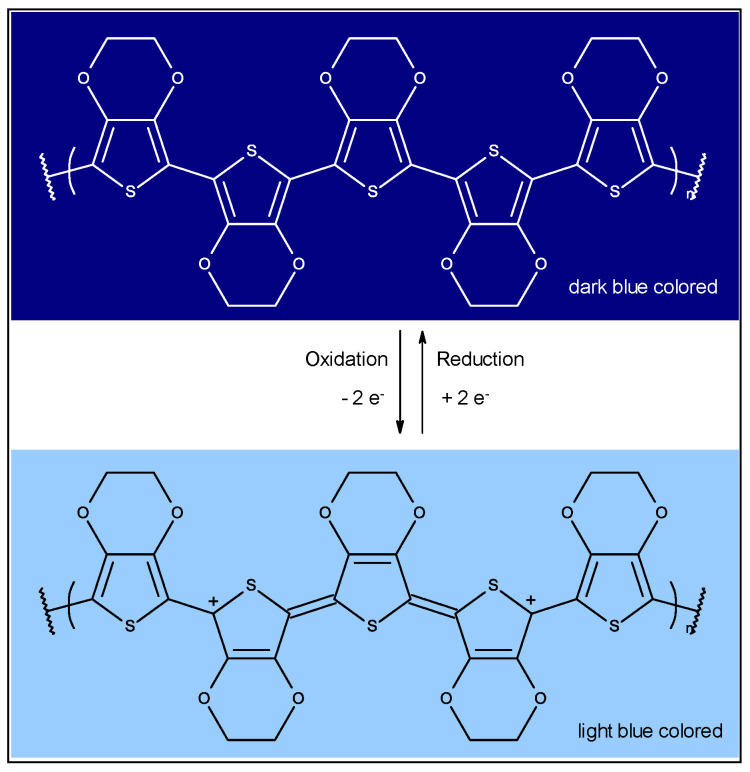
Redox scheme for PEDOT: PSS electrode (for clarification reasons the section shows five conjugated monomer units).

Besides inorganic thin layers, organic and metal-organic compounds can also exhibit electrochromism. These compounds are, e.g., small molecules like benzothiadiazole fluorophores, bipyridylium salts (also called viologens), and electrically conductive polymers like derivatives of polyaniline (PAni) and polythiophene (PT) [15,16,17,18,19,20,21,22]. These polymers exist in a broad variety of oxidation states, which all appear in a different color, e.g. for PAni three different colored states are known: the colorless leucoemeraldine, green emeraldine and dark blue pernigraniline [18,23]. Poly-3,4-ethylenedioxythiophene polystyrene sulfonate (PEDOT:PSS) exists in two states. The oxidized cationic state it is light blue colored and semi-transparent, and when reduced to the neutral state it is dark blue (Figure 1) [24].

Compared to inorganic compounds, the presented electrochromic polymers offer some advantages. They are flexible, easily processable, and can act both as an electrode and electrochromic layer, which reduces the necessary number of layers in the cell. PEDOT:PSS has attracted much attention as a conducting polymer in various application fields due to its high conductivity, and since it is easily processable, as a water-based dispersion [25,26,27,28].

**Figure 2:**
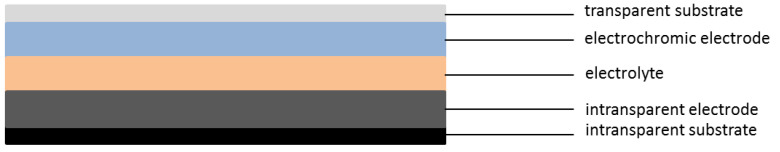
Schematic built-up of an electrochromic device with an electrochromic electrode.

The principle built-up of an electrochromic device is shown in Figure 2. Between two electrodes, of which one carries the electrochromic material, an electrolyte is sandwiched. The electrolyte layer must provide the necessary charge mobility to reduce and oxidize the electrochromic layer, thus enabling the color change. 

For an optimal performance, the choice of electrolyte is crucial, because it acts both as an ionic conductor between the electrodes and as a reservoir for charge carriers moving into and out of the electrochromic layer [29]. Small molecules or ionic electrolytes dissolved in any solvent are not practicable for porous substrates such as fabrics due to evaporation. Therefore, different polymer electrolyte types have been investigated in the past. Among the liquid polymer electrolytes, poly diallyldimethylammonium chloride (PDADMAC) has been investigated [30]. An ionic gel polymer electrolyte for electrochromic cells was presented by Kobayashi and co-workers using polyvinyl butyral as host polymer and tetrabutylammonium perchlorate as ionic conductor [31]. Also, natural polymers based on starch, cellulose, and chitosan were investigated as a host matrix for electrolytes not only in electrochromic cells, but also for assembling capacitors, batteries, and solar cells [32,33,34]. An alternative host matrix for electrolytes used in these applications is gelatin [35]. Gelatin is a mixture of different animal proteins, mainly denatured collagen derived from cattle and pig connective tissue. After immersing gelatin in water, it swells and dissolves, depending on its production process and exact composition when heated above 50 °C. Mixtures of glycerol and gelatin are known to keep water molecules in the film due to the hygroscopic effect of glycerol. Furthermore, the addition of glycerol increases the water vapor barrier of the film [36].

Most electrochromic devices are fabricated on rigid substrates. In the last decade electrochromic devices were also produced on flexible substrates such as elastomeric substrates, textiles and paper [35,36,37,38,39]. The conductive layer can be combined with flexible polydimethylsiloxane, which allows the use of tungsten oxide as electrochromic material despite its brittleness.

Colored and color changing fabrics are sometimes desired features not only in the design of clothing, but also in technical applications, where a color change can be even more important for displaying sensed information or for camouflage purposes [29]. A change in the state of system can be displayed on an electrochromic display. To display more than only binary information (on/off), a pixel matrix with individually addressable pixels is necessary. With such a matrix, words or numbers can be displayed. The availability of the pixels allows for the flexible display of any information, and a predefinition through printing or coating is not necessary.

Several approaches for combination of electrochromic devices and textiles have been presented in the past years. Notably, Moretti and co-workers have reduced the necessary layers for flexible electrochromic devices [40,41].

**Figure 3:**
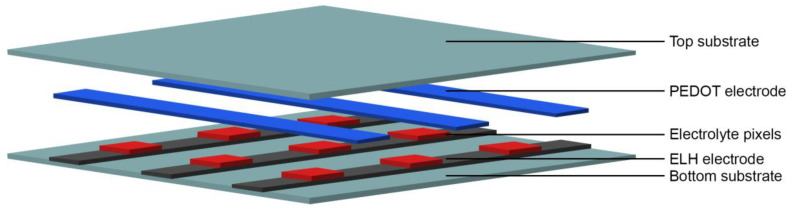
Built-up of a 3 × 3 electrochromic matrix with switchable pixels.

In this work, textile-based electrochromic cells with two different gel-based electrolytes are presented. The color-changing material herein is PEDOT:PSS, which also acts as the front electrode. For the presented textile-based electrochromic devices, a five-layer assembly is necessary (Figure 3).

In addition to the electrolyte pixel, an insulating material can be applied in the same layer using masking technology. This guarantees that only the desired pixels are switched on when a voltage is supplied. Furthermore, the insulating layer can be filled with inorganic pigments, providing an additional and persisting color.

## 2. Materials and Methods

### 2.1. Preparation of Gel Electrolytes

#### 2.1.1. Electrolyte E1

Electrolyte E1 was prepared by mixing 10.0 g Polydiallyldimethylammonium chloride (PDADMAC) solution (20% in water, Sigma Aldrich, Steinheim, Germany) and 1.0 g titanium dioxide (Kronos 2360, Leverkusen, Germany). In a second flask 0.2 g sodium alginate (VWR, Leuven, Belgium) were dissolved at 60 °C in 10 ml deionized water. Both solutions were mixed and stirred thoroughly resulting in a viscous liquid.

#### 2.1.2. Electrolyte E2

To a prepared mixture of 1.0 g gelatin powder (microbiology grade, Merck, Darmstadt, Germany) in 14 mL glycerol (ACS reagent grade, Sigma-Aldrich, Steinheim, Germany), 6.0 mL deionized water, 0.5 g titanium dioxide in rutile modification (Kronos 2360, Leverkusen, Germany), and 0.3 g calcium chloride (97%, Carl Roth, Karlsruhe, Germany) were added. After homogenization at room temperature, the mixture was swelled for one hour. Then the mixture was slowly heated up under stirring to 60 °C for 1 min. The electrolyte can be stored at RT for at least one month and should be warmed prior to use.

### 2.2. Substrates and Equipment

**Figure 4:**
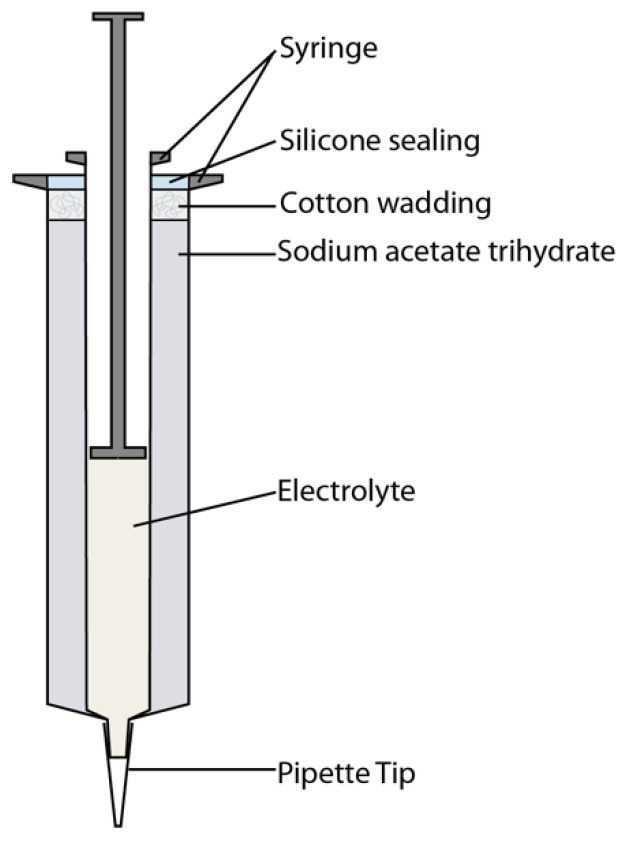
Double-walled syringe for the application of electrolyte E2.

The textile substrates were kindly provided by Schmitz-Werke, Emsdetten, Germany. The coatings were performed on a polyester (PES) plain weave fabric (200 g/m²) and a semi-transparent polyester rip-stop fabric (120 g/m²). Further, the fabrics were pre-coated with the polyurethane dispersion Tubicoat MEA (CHT R. Beitlich, Tübingen, Germany) to avoid bleeding blurs of the coated and sprayed electrode layers using a 40-µm roller bar. Polyethylene terephthalate (PET) film with a thickness of 25 µm was provided by Inoviscoat GmbH, Monheim am Rhein, Germany. A transparent polyethylene polyester membrane (PEPES) was provided by Sympatex, Unterföhring, Germany. Roller bar coatings were performed with a 40 µm gap size bar. Spray coating was performed with a Revell airbrush pistol. Electrolyte E1 was applied with a pipette whereas the electrolyte E2 was applied with a heated, double-walled syringe (see Figure 4). 

The space between the walls was filled with a supersaturated sodium acetate trihydrate solution. The exothermic crystallization of sodium acetate trihydrate solution provides enough heat to ensure, that the electrolyte remains liquid during the application [42]. Since the crystallization is a reversible reaction, the applicator is reusable after reliquification of the sodium acetate trihydrate. The substrates were laminated together using a colorless Toolcraft spray adhesive from Conrad Electronic SE (Hirschau, Germany). 

### 2.3. Coating of Electrode Layers

Two methods were investigated and compared for the production of the electrode layers: 1) roller bar coating and 2) spray coating. In the case of roller bar coating, the dispersions were used as received after stirring. For spray coating, the dispersions were diluted with isopropanol (Carl Roth, Karlsruhe, Germany) in a mass ratio of 1:1. 

For the bottom electrode, stripes of Tubicoat ELH (CHT R. Beitlich, Tübingen, Germany) were coated onto the textile substrate. The length of the stripes was 100 mm and they were 25 mm width. A roller bar with a gap size of 40 µm was used, the distance of 2.5 cm between the stripes was fixed with a removable tape. The coating was dried at 100 °C for 120 s and annealed afterwards at 150 °C for 300 s.

The transparent top layer serves both as the electrochromic layer and counter electrode. PEDOT:PSS (Clevios S V4, Hereaus, Leverkusen, Germany) was coated with a roller bar (40 µm gap size) onto the substrate, which was in this case also the semi-transparent PES rip-stop fabric. PEDOT:PSS was also spray coated on PET film. The coating was dried at 100 °C for 120 s. In the second approach, it was spray-coated with a mixture of PEDOT:PSS and isopropanol onto the PEPES membrane.

To realize the patterning, a stencil prepared beforehand was securely placed on the substrate. Individual stencils were prepared for the different layers to produce a 5 × 5-pixel ECD matrix. For the counter electrode, a mask with up to five horizontal stripes (6 mm width, 100 mm length, 6 mm apart), a stencil with similar vertical stripes, with the same measurements, was prepared for the electrochromic electrode. A shadow mask for the masking layer was prepared accordingly. In order to achieve a thin, evenly coated substrate, the material was sprayed for 5 s from a distance of 20 cm. The coating was dried in the oven for 60 s at a temperature of 100 °C.

### 2.4. Insulating Layer for Electrochromic Matrices

For the insulating layer 9.50 g Tubicoat MEA (CHT R. Beitlich, Tübingen, Germany) were mixed with 0.5 g titanium dioxide (Kronos 2360, Leverkusen, Germany) and 10.0 g isopropanol (Carl Roth, Karlsruhe, Germany). The dispersion was spray coated on top of the counter electrode stripes using a stencil with spaces for the electrolyte. For stencils see Appendix A.

### 2.5. Assembly of Electrochromic Device

**Figure 5:**
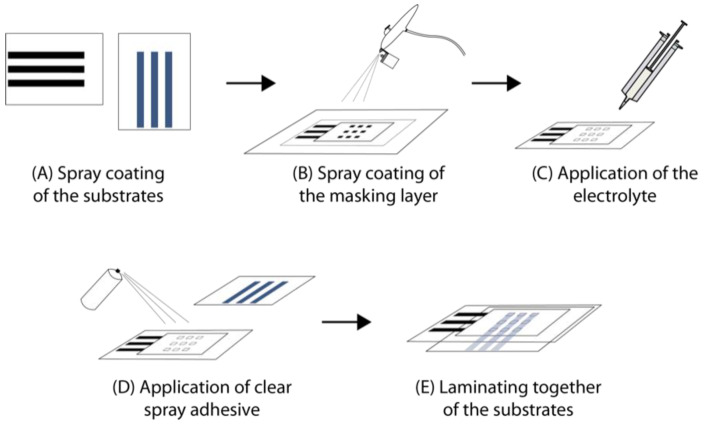
Assembling steps of electrochromic devices by spray coating process.

**Figure A1:**
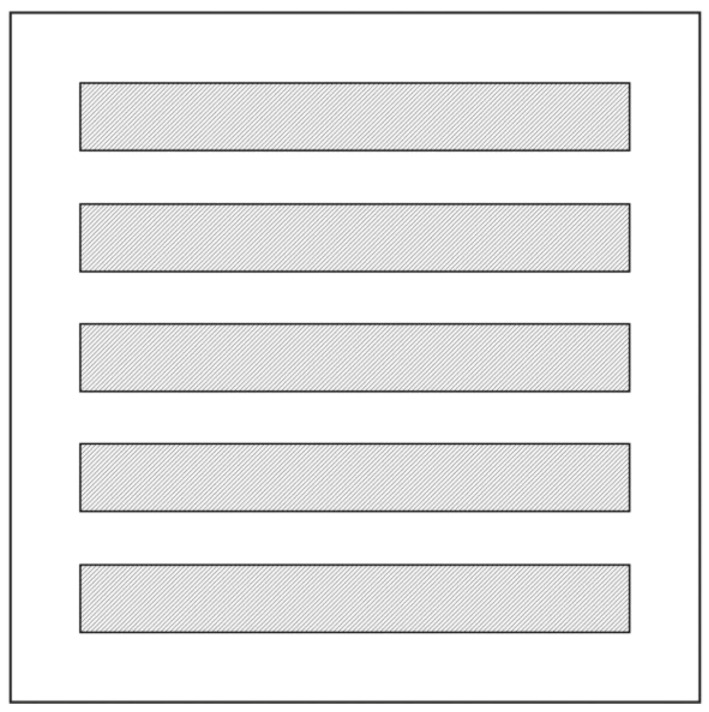
Stencil for the 5 × 5 ECD pixel electrodes.

The assembly steps of electrochromic cells by spray coating is described in Figure 5. Stripes of conductive dispersions were coated or sprayed on the substrates using a stencil (Figure A1). PEDOT:PSS as electrochromic materials was deposited on PEPES and Tubicoat ELH on the textile substrate (A). The insulating layer was applied on top of the counter electrode via spray coating using the cut out squares from stencil A2 as a masks (B).

Afterwards the electrolyte E2 was applied with the help of a heated applicator, extending the time window for application, which consisted of a double walled syringe (C). The rest of the fabric was covered with stencil A2. After gelling of the electrolyte, a spray adhesive was applied on the prepared half-cell. The cut-out squares from the stencil A2 were used to protect the electrolyte layer (D). The electrochromic electrode was laminated on top of the spray adhesive (E).

### 2.6. Functionality Tests of Assembled Electrochromic Cells

Electrochromic cells with PDADMAC alginate gelled electrolyte (E1) were assembled with roller bar coated top and bottom electrodes. Three cells were prepared with this electrolyte. The first cell was switched 25 times between “on” and “off”. The second cell was switched “on” and the power supply unit was turned off after darkening of the PEDOT:PSS layer. The third cell was switched “on” and “off” directly after assembling and then again after two hours for the second time.

Similar to the electrochromic cells with PDADMAC as electrolyte, the cells with the electrolyte E2 were investigated. Individual pixels of electrochromic matrices were switched on and off separately. Over a period of one year the 5 × 5 electrochromic matrix was switched and off on a regular basis to evaluate its functionality.

### 2.7. Characterization of Electrochromic Devices

The prepared electrochromic devices were characterized with regard to their electrical and chromatic properties. A HCS-3404 power supply unit from Manson Engineering was used to power the electrochromic cells. The switching time was determined by measuring the time needed to a fully colored state. Scanning electron micrographs were obtained with TM4000Plus from Hitachi and UV/Vis spectra were recorded with a UV-2600 spectrometer from Shimadzu. Chromaticity was measured with a Datacolor 400 chromatometer from Datacolor using D65 light with a 10-degree observer. The color contrast ΔE* is given by the Euclidean distance between the color coordinates of the different redox states and was calculated in the CIELAB color space (see in Appendix B), which well adapts the vision of the human eye [43]. The respective formula is:

(1) $$\Delta E^{*}=\sqrt{\Delta L^{{*}^{2}}+\;\Delta a^{{*}^{2}}+\Delta b^{{*}^{2}}}$$

I/V-data were acquired using a power source, which was controlled by a LabView program. Data were acquired by two point measurements between –5 and +5 V.

## 3. Results and Discussion

### 3.1. Electrochromic Cells with Electrolyte E1 in Combination with Roller Bar Coated Electrodes

Directly after assembling, all cells showed the same characteristics. The switching time of the freshly prepared cells was 4 s. Switching between oxidized and reduced state of PEDOT:PSS occurred at a voltage of 2.5 V. The switching time for the first cell remained at 4 s after 25 cycles. The second cell sustained the reduced state for ten minutes, from the moment the oxidation process was observable. The switching time of the third cell increased to 11 s at a voltage of 2.5 V after two hours indicating a loss of functionality. Textile substrates are permeable for air and water moisture, thus water of the electrolyte evaporated. The alginate gel was not capable to hold back enough moisture, and therefore the electrochromic cell stopped working within a few hours. This was supported by the fact that the dried electrolyte was found in small crumbs distributed on the electrodes when the cell was disassembled. This explains, why the charge carriers could not be transported in the dried PDADMAC alginate gel. Nevertheless the cell was roughly characterized. Even though PDADMAC can be used as an electrolyte itself, titanium dioxide pigments were added to enhance the contrast between the oxidized and the reduced state of PEDOT:PSS. Overall, the prepared electrolyte works for electrochromic cells, though due to the lack of encapsulation, which would result in the loss of textile haptics, it is not suitable for textile-based electrochromic cells.

### 3.2. Comparison of Electrical Resistance and Surface Morphology on Different Substrates

All electrochromic devices were fabricated method-consistently. A spray coated PEDOT:PSS electrode was always combined with spray coated counter electrode and a roller-bar coated PEDOT:PSS electrode was combined with a roller-bar coated electrode, respectively.

**Figure 6:**
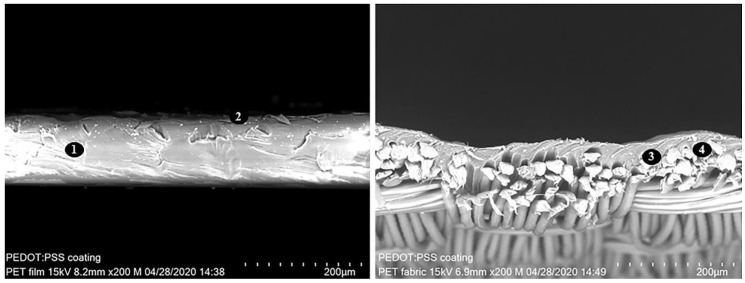
SEM crosscut micrographs of PEDOT:PSS (2) roller-bar coated PET film (1) (left) and PET fabric (right). The coated film is flat, whereas the fabric is coated unevenly with thick (3) and thin spots (4) on the surface.

**Figure 7:**
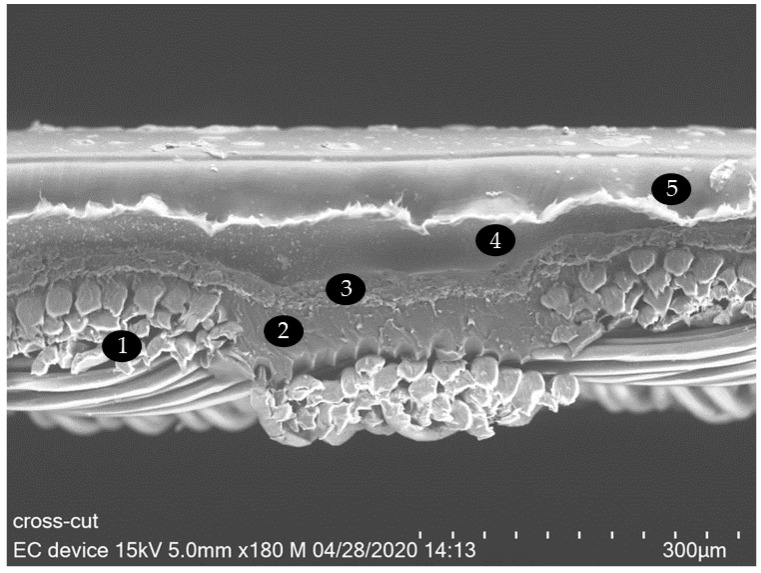
SEM crosscut micrograph of an electrochromic device with 1—PES fabric, 2—polyurethane pre-coating, 3—counter electrode, 4—electrolyte with titanium dioxide, 5—top substrate with PEDOT:PSS layer.

In contrast to coatings on PET film, roller-bar coating on PES fabric did not result in even and smooth layers. This is due to the unevenness of the woven material on a microscopic scale (see Figure 6 and Figure 7).

The coating dispersion accumulated in the cavities of the fabric, leading to a thicker layer than on top of the yarn intersections. Also, patterning options were quite limited with this technique. Patterning with masks or adhesive tape resulted in uneven layers and therefore in a non-uniform electrical resistance of the respective layer. Especially at the edges of the mask or tapes, the layer became thicker. The sheet resistance of roller-bar coated Tubicoat ELH on the rip-stop fabric was 50 Ω_sq_, whereas the coated PEDOT:PSS layer on PES rip-stop fabric was 250 Ω_sq_.

Compared to roller-bar coating spray coating is not a direct method. The use of spray coating allowed the use of stencils with different patterns. However, the herein used commercially available conductive dispersions Clevios S V4 for the electrochromic electrode and Tubicoat ELH for the counter electrode were too viscous for spray application. For that reason, both dispersions were diluted with isopropanol, which resulted in a sprayable dispersion. Isopropanol has got a low boiling point, which allowed a fast drying of the sprayed layers. The sheet resistance of a spray coated diluted Tubicoat ELH layer on the rip-stop fabric was 130 Ω_sq_, whereas the spray coated PEDOT:PSS electrode on the PEPES membrane had a sheet resistance of 500 Ω_sq_. 

The switching time of electrochromic devices is known to be influenced by the speed of electron diffusion into the electrochromic layer. The diffusion rate and therefore also the switching speed is higher for thinner layers [44]. The lower conductive PEDOT:PSS layer is favored for a faster switching electrochromic device. The conductivity of the PEDOT:PSS layer is controlled by the application method and dilution of the dispersion. Nevertheless, it was not possible to spray coat the commercially available PEDOT:PSS dispersion due to its high viscosity and the viscosity of diluted PEDOT:PSS was too low for roller-bar coating. Therefore, no direct comparison was possible.

### 3.3. Comparision of Electrolytes

The electrolytes are chemically different. However, both electrolytes are based on natural gels. Electrolyte E1 contains alginate and electrolyte E2 is based on gelatin. Both electrolytes contain titanium dioxide as ion storage. Furthermore, titanium dioxide in the electrolyte enhances the contrast of the electrochromic layer. The ionic compound in electrolyte E1 is PDADMAC, which is a well-known water soluble electrolyte, whereas calcium chloride was chosen in electrolyte E2 as ionic conductor.

Initially, textile-based electrochromic devices with both electrolytes work at low voltages (2.5 V for electrolyte E1 and 2.0 V for electrolyte E2). The devices have switching times of 4 s for electrolyte E1 and 2 s for electrolyte E2. This already indicates, that the gelatin-based electrolyte has a higher performance than the alginate based electrolyte.

Electrolyte E2 shows also a clear better long-term performance in the presented electrochromic devices. The devices with this electrolyte are stable for more than one year, whereas devices with electrolyte E1 showed a reduced functionality after two hours due to drying of the electrolyte. In electrolyte E1 PDADMAC lost its functionality due to the evaporation of water. The presence of water or moisture in the electrolyte is crucial for the transportation of charge carriers into or out of the electrochromic layer. Therefore, the addition of glycerol as a hygroscopic compound in electrolyte E2 resulted in the long-term stability and functionality of the electrochromic device.

### 3.4. Electrochromic Cells with Electrolyte E2

A textile-based electrochromic cell was assembled with PEDOT:PSS as electrochromic layer and the gelatin-based electrolyte E2. A SEM micrograph of the crosscut electrochromic cell is shown in Figure 7. The PEDOT:PSS layer cannot be resolved in the SEM micrographs due to its too low thickness.

**Figure 8:**
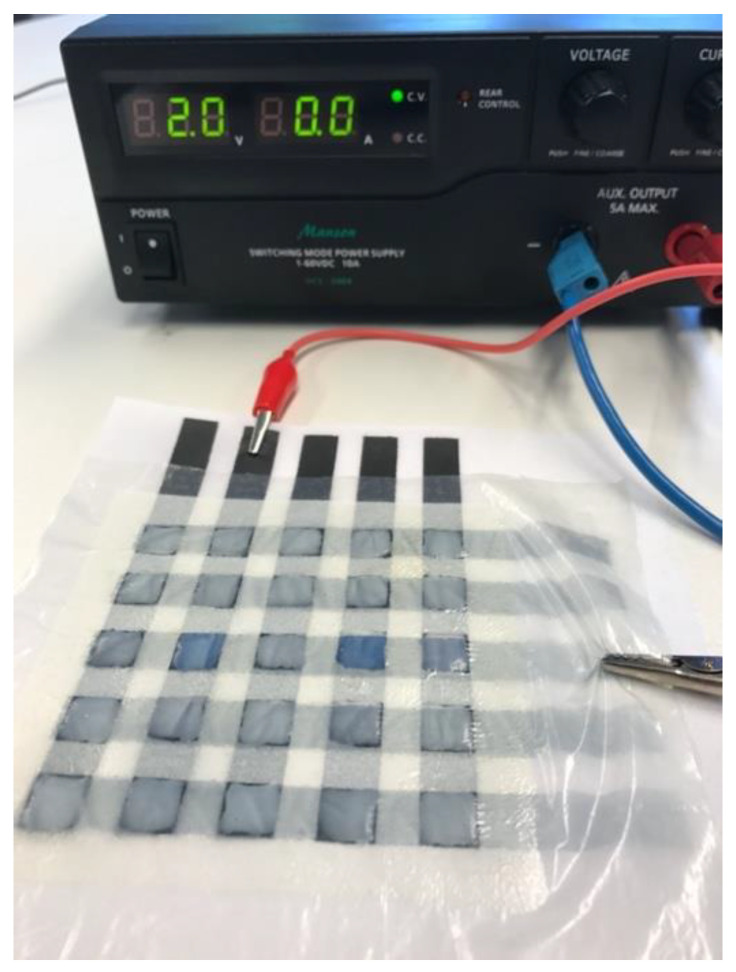
5 × 5 electrochromic device where two pixels have been switched at 2.0 V as indicated on power supply unit. As it can be seen the pixels can be switched individually.

The cells are flexible and bendable. Further, the textile-based devices could also be creased to some extent. The switching time directly after assembling was 2 s at a voltage of 2.0 V. Moreover, the switching time and voltage remained constant after 25 cycles. Crumpled and re-flattened devices were still working, while the switching time and voltage did not change. The electrolyte was quite stable and held back moisture over an extended period of time. Even after one year, the cell could still be switched on and off. After one year, the switching time increased and was found to be at around 4 s at 2.0 V. A 5 × 5 electrochromic matrix with this configuration is shown in Figure 8.

The color contrast ΔE* was calculated according to formula 1 to be 21.25 for a pixel of the 5 × 5 pixel electrochromic device. For the human eye the color changes from a very light blue shade to a dark blue color, as one can also see in Figure 8. The color of a reduced PEDOT:PSS pixel sustains for a moment, and therefore two pixels in this figure are shown in the colored state.

**Figure 9:**
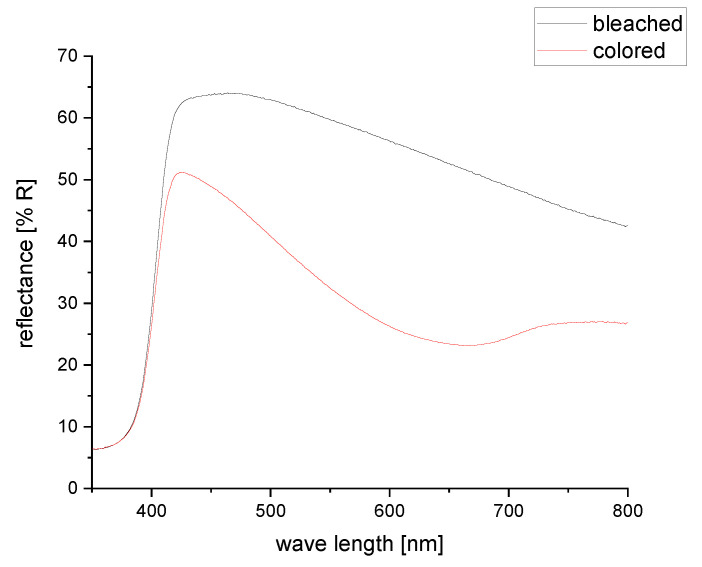
Reflectance spectra of electrochromic cell in bleached and colored states.

Coloration efficiency is an important characteristic for electrochromic devices. It is calculated using the transmittance of bleached and colored state. However, in this case no transmittance in the visible range can be measured due to the high reflection and light scattering on titanium dioxide particles in the electrolyte and the high absorption of light on the black counter electrode. Therefore, reflectance spectra of bleached and colored states were measured in the visible light range between 350 and 800 nm (Figure 9).

The diffuse reflectance of the bleached device in the visible range is between 50% and 60%, which can be explained by the high amount of titanium dioxide in the electrolyte. The reflection decreases in the higher wavelengths, which is explained by the light blue shimmer of the electrochromic cell and by the absorption of light in the PEDOT:PSS layer on the front electrode. When the device is switched on, the overall reflection decreases. The maximum at 425 nm is now at 51% reflection and the reflection decreases steeper until a minimum reflectance of 23% is found around 660 nm. This spectrum coincides with the deep blue color of PEDOT:PSS in the reduced state.

**Figure 10:**
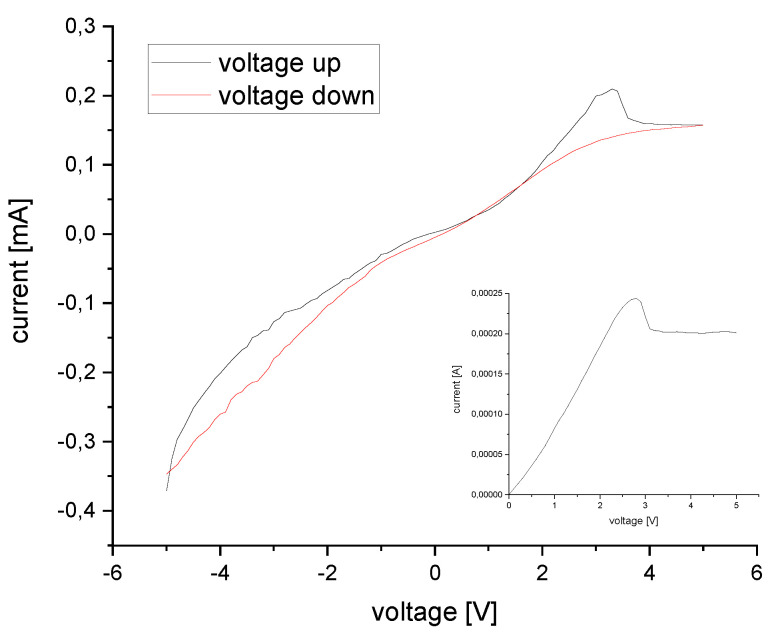
Voltage-current data for one cycle from −5 to +5 V and from 0 to +5 V (inlay).

The electrochromic device shows an ohmic behavior between 0 and +2.8 V, when it is measured from 0 to +5 V (see inlay in Figure 10). The total resistance of the device was calculated to be around 15 kΩ. When the PEDOT:PSS is reduced completely to its insulting state, the current drops and remains constant above 3.1 V. Starting the measurement at −5 V, results in a slightly different behavior (Figure 10). Between –3 V and +1 V, the I–V-curve is linear, which means that the resistance of the device is constant. The current increases steeper above +1 V, but again the increase is linear. When the voltage is reduced, the current decreases only slightly and is non-ohmic until the voltage reaches +2 V, then a linear decrease of the current is observed, which becomes steeper at –1 V. A small hysteresis is observed at the start and end point –5 V. The final current is a little bit higher than the current in the beginning (Figure 10).

The fabricated textile ECD prototype has a 5 × 5-pixel matrix (Figure 7). So far, it is a simple display which is patternable. It has the further potential to be integrated into an autonomously actuated system in the future. Color can be switched on and off at the (active) addressable pixels. 

## 4. Summary and Conclusions

The successful fabrication of a textile integrated ECD with individually addressable pixels for the controllable ability to change the display of patterns and coloration is demonstrated. Extension and miniaturization of the pixel matrix allow a flexible display for information or camouflage purposes. A moisture-retaining agent in the electrolyte ensures that the electrolyte does not dry out and the electrochromic cell retains its functionality. The white backing of the pixel electrodes through the opaque white pigmentation of the electrolyte enhances the color contrast of the pixel electrode in the layer above. The electrochemical behavior of this electrochromic cell is not yet well understood and must be further investigated.

## Figures and Tables

**Figure A2:**
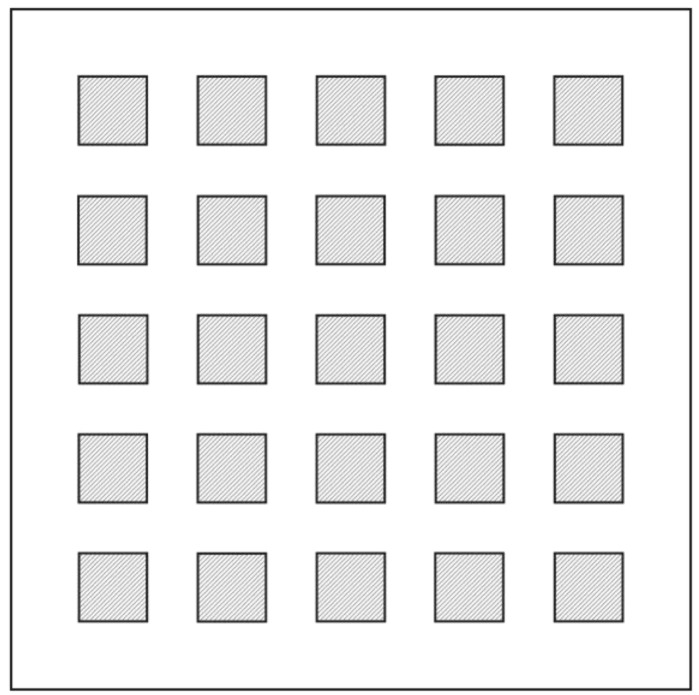
Stencil for 5 × 5 ECD electrolyte layer.

## Appendix A. Preparation of stencils

All stencils were cut out of PET film. The grey shaded areas were cut out. The cut out squares from stencil A2 were used to mask the electrolyte pixels when spraying the polyurethane masking layer.

## Appendix B. Color Contrast

**Table A1:** Chromaticity of EC devices in CIELAB color space (light source: D65, 10 degree observer).

	In Oxidized State	In Reduced State
L*	80.28	65.48
a*	−3.61	−5.83
b*	−2.18	−17.27

According to formula 1: ΔE* = 21.25.
